# Bonding Strategies for Zirconia Fixed Restorations: A Scoping Review of Surface Treatments, Cementation Protocols, and Long-Term Durability

**DOI:** 10.3390/biomimetics10090632

**Published:** 2025-09-19

**Authors:** Iulian-Costin Lupu, Monica Silvia Tatarciuc, Anca Mihaela Vitalariu, Livia Bobu, Diana Antonela Diaconu, Roxana-Ionela Vasluianu, Ovidiu Stamatin, Cosmin Ionut Cretu, Ana Maria Dima

**Affiliations:** 1 Department of Prosthodontics, Faculty of Dental Medicine, “Grigore T. Popa” University of Medicine and Pharmacy, 700115 Iasi, Romania; iulian.lupu@umfiasi.ro (I.-C.L.); ovidiu.stamatin@umfiasi.ro (O.S.); cosmin.cretu@umfiasi.ro (C.I.C.); 2 Department of Dental Prosthesis Technology, Faculty of Dental Medicine, “Grigore T. Popa” University of Medicine and Pharmacy, 700115 Iasi, Romania; tatarciucm@yahoo.com (M.S.T.); anca.vitalariu@umfiasi.ro (A.M.V.); antonela.diaconu@umfiasi.ro (D.A.D.); 3 Department of Surgicals, Faculty of Dental Medicine, “Grigore T. Popa” University of Medicine and Pharmacy, 700115 Iasi, Romania; livia.bobu@umfiasi.ro; 4 Independent Researcher, 700115 Iasi, Romania; amadi2024@proton.me

**Keywords:** zirconia, bonded restorations, adhesive technology, 10-methacryloyloxydecyl dihydrogen phosphate primer, surface treatment, shear bond strength, aging protocol, thermocycling, prosthodontics, biomimetics

## Abstract

Zirconia’s superior mechanical properties and biocompatibility have made it a cornerstone of modern prosthodontics, yet achieving durable biomimetic bonding to tooth structure remains a challenge. This scoping review synthesizes evidence on bonding strategies for zirconia-based fixed dental prostheses (FDPs), evaluating surface treatments, cementation protocols, and long-term performance. Following PRISMA-ScR guidelines, 18 studies from PubMed, Scopus, Web of Science, and Embase were thoroughly analyzed. Key findings indicate that tribochemical silica coating (e.g., Rocatec™) combined with 10-methacryloyloxydecyl dihydrogen phosphate (MDP)-based primers (e.g., Panavia V5) is associated with the highest bond strengths (>40 MPa) and exceptional clinical survival rates (e.g., >95% at 15 years for resin-bonded FDPs). These combined mechanical–chemical strategies can be viewed as an attempt to create a biomimetic, hybrid interface akin to the natural enamel–dentin junction. Additively manufactured zirconia exhibits inferior bonding compared to milled counterparts, while ethyl cellulose coatings applied to the bonding surface effectively prevent contamination from saliva and moisture during intraoral try-in procedures. However, heterogeneous testing protocols and limited long-term clinical data highlight the need for standardized aging models and randomized trials. This review consolidates current evidence, offering clinically actionable recommendations through a biomimetic lens while identifying critical gaps for future research.

## 1. Introduction

**Figure 1:**
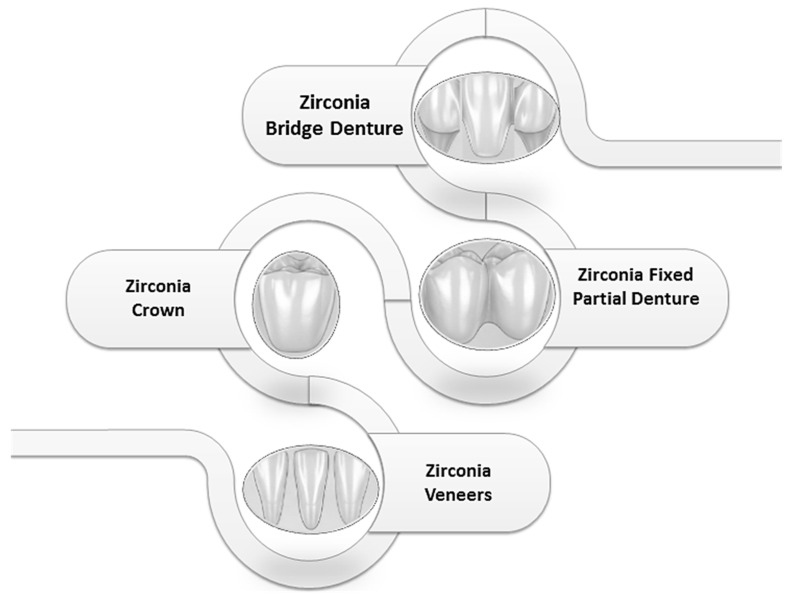
Zirconia Applications in Prosthodontics.

The rise in zirconia-based restorations marks a paradigm shift in modern prosthodontics, driven by their unparalleled biomechanical properties, biocompatibility, and esthetic potential [1,2,3,4,5,6]. As a polycrystalline ceramic devoid of a glassy phase, zirconia (ZrO_2_) derives its strength from transformation toughening, a mechanism that resists crack propagation, making it ideal for high-load applications such as crowns, fixed dental prostheses (FDPs), and resin-bonded restorations [7,8,9,10]. The figure below represents the main prosthodontic applications of zirconia in dental medicine: crowns, bridge, veneers, and fixed dentures (Figure 1).

However, its chemical inertness and absence of silica content challenge conventional adhesive protocols, which rely on micromechanical retention (e.g., acid etching) and chemical bonding (e.g., silane coupling agents) [11,12]. This inherent limitation has spurred extensive research into surface modifications, bioactive primers, and resin cements to achieve durable adhesion, yet debonding remains a leading cause of failure, with reported rates of 5–15% for resin-bonded FDPs over five years [13,14].

The evolution of zirconia bonding strategies reflects the convergence of material science and biomimetic principles [15]. The ultimate goal is to create a durable, stress-resilient tooth–restoration interface that mimics the biological excellence of the natural enamel–dentin junction (EDJ), a functionally graded zone that seamlessly integrates two dissimilar tissues [16,17]. Early approaches depended on macromechanical retention through retentive preparation designs, but the demand for minimally invasive prosthetics necessitated innovations in surface treatments and cement chemistry [18,19,20]. Surface roughening treatments, particularly airborne-particle abrasion (sandblasting), are a critically effective and widely validated method for enhancing the bond strength of zirconia-based dental restorations by increasing micromechanical retention and, in some cases, inducing beneficial compressive surface stresses, though the specific protocol must be optimized to avoid excessive surface damage that can compromise flexural strength [21,22,23,24,25,26,27]. The introduction of 10-methacryloyloxydecyl dihydrogen phosphate (MDP), a monomer capable of chemically bonding to zirconia’s oxide layer, revolutionized adhesion by complementing micromechanical retention with stable chemical interactions [28,29,30,31,32]. Despite this advancement, the proliferation of zirconia formulations (3Y-TZP, 4Y-PSZ, 5Y-PSZ) and manufacturing techniques (e.g., milling, additive manufacturing) has introduced new variables that influence bonding efficacy [33,34]. For instance, higher yttria content (5Y-PSZ) enhances translucency but reduces flexural strength, while subtractive manufacturing yields denser interfaces than 3D-printed zirconia, impacting resin infiltration and bond durability [35,36,37,38].

Contemporary bonding protocols now encompass three main dimensions:

1. Surface pretreatments, such as tribochemical silica coating (e.g., Rocatec™), nanostructured alumina deposition, or laser ablation, which modify surface energy and topography, effectively creating a micro-rough, silica-rich surface that biomimics the retentive morphology of etched enamel [39,40];
2. Primer and cement chemistry, including MDP-based primers and self-adhesive resins (e.g., Panavia V5), which optimize chemical adhesion, forming molecular bridges that emulate the organic–inorganic bonding of the hybrid layer in dentin [41,42];
3. Aging resistance, where thermocycling and mechanical fatigue critically influence long-term performance [43,44,45].
4. Yet clinical adoption remains inconsistent due to conflicting evidence, technique sensitivity, and a lack of consensus on optimal protocols [46]. For example, while airborne-particle abrasion with alumina enhances bond strength, excessive pressure may compromise the mechanical integrity of translucent zirconia [47,48,49,50]. Similarly, the efficacy of MDP primers varies with zirconia composition and surface treatment, underscoring the need for standardized guidelines [51,52,53,54,55].

**Figure 2:**
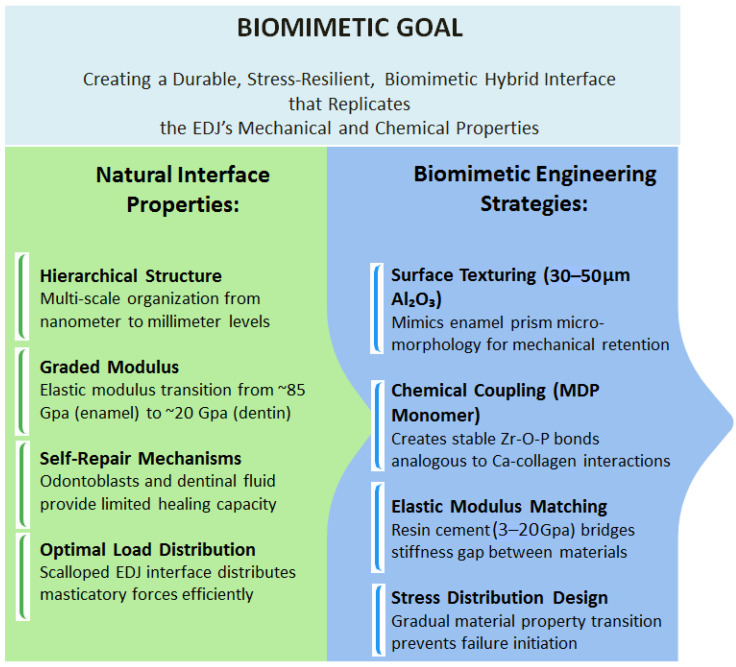
Biomimetic bonding interface analogy.

This scoping review synthesizes a decade of evidence (2015–2025) to evaluate the interplay between zirconia material science, bonding protocols, and clinical outcomes. Specifically, it aims to map evidence on efficacy of bonding techniques for zirconia-based fixed dental prosthesis. The imperative for optimized bonding extends beyond preventing debonding; it is increasingly important to achieving a biomimetic restoration. A poor interface risks microleakage, secondary caries, pulpitis, and catastrophic failure, while overly aggressive surface treatments may compromise the material’s integrity. As zirconia continues to dominate restorative dentistry, this review maps current evidence, identifies knowledge gaps, and offers evidence-based recommendations to enhance the longevity and predictability of zirconia restorations through biomimetically informed strategies. The figure below represents an analogy of biomimetic bonding interface (Figure 2).

## 2. Materials and Methods

### 2.1. PRISMA-ScR Compliance

This scoping review adhered to the guidelines outlined by Tricco et al., 2018, in the PRISMA Extension for Scoping Reviews methodology (PRISMA-ScR) [56]. The full checklist is listed in the Supplementary File S1.

### 2.2. Objective

This scoping review evaluated the evidence on optimal bonding strategies for zirconia fixed restorations, focusing on surface treatments, cementation protocols, and their long-term durability.

### 2.3. Search Strategy

To ensure methodological rigor while maintaining breadth, this scoping review systematically examined the literature from PubMed, Scopus, Web of Science, and Embase for zirconia, dental bonding, and fixed prostheses (full syntax provided in Supplementary File S2). Searches were conducted on July 2025, capturing advancements in zirconia bonding technologies. The search spanned January 2015–June 2025 and was limited to English-language studies.

The strategy prioritized evidence on adhesive performance while identifying gaps in long-term clinical validation. This systematic approach guaranteed that the review identified the most pertinent and credible evidence while maintaining transparency for reproducibility. By employing a broad yet focused search across major databases, this strategy effectively synthesizes existing knowledge and highlights gaps for future research.

### 2.4. Eligibility Criteria

This review focused on peer-reviewed studies published in English between 2015 and June 2025. It includes comparative investigations evaluating bonding strategies for zirconia restorations, encompassing both laboratory studies with standardized aging protocols and clinical studies with survival data. Systematic reviews were consulted for contextual background but excluded from primary analysis.

To ensure robust comparisons, selected studies had to report quantitative outcomes including

- Bond strength measurements (shear/microtensile in MPa);
- Clinical performance metrics (survival/debonding rates);
- Durability assessments (thermocycling, water storage, or chewing simulation results);
- Failure mode analyses (adhesive/cohesive/mixed fractures).

The scope specifically examined permanent zirconia restorations, including crowns, fixed dental prostheses, and resin-bonded bridges, while excluding temporary prostheses. Table 1 details the complete eligibility framework with scientific rationale.

### 2.5. Literature Screening and Prioritization

The literature selection was recorded following the PRISMA-ScR protocol flow diagram. To ensure thorough and methodically sound inclusion of relevant studies, this scoping review employed a structured, multi-step screening approach. The preliminary database searches retrieved 4058 records, reflecting widespread scientific interest in zirconia bonding methods for fixed dental prostheses. To streamline further screening, 253 records without a DOI were excluded, as they failed to meet fundamental eligibility criteria.

A thorough deduplication process was then conducted, eliminating 2053 duplicate records using a predefined method. This was executed in Microsoft Excel with a tailored formula to guarantee precise and accurate record handling.

Screening of the remaining 1752 records, based on titles and abstracts, was performed in accordance with the inclusion criteria. Exclusions (*n* = 1727) were made for studies that (1) examined non-zirconia materials or adhesion methods for dentures; (2) assessed pediatric crowns or lacked a clear bonding methodology (e.g., insufficient details on surface treatment or cementation); or (3) involved animal testing or were published in non-English languages. As a result, 25 studies remained for full-text evaluation.

The final 25 studies were subjected to a strict eligibility assessment. Two reviewers, A.M.D. and O.S., independently evaluated each study, with any disagreements resolved through discussion. Seven records were excluded due to the following reasons:

- Lack of bond strength measurements;
- Absence of an aging protocol;
- No comparison between MDP and the defined* silica-based approaches;
- Animal or in vitro studies lacking clinical applicability;
- Not focused on zirconia bonding;
- Review articles or editorials;
- No standardized bonding protocol.

* For the purposes of this review, “silica-based approaches” were defined as any surface treatment method that deposits or creates a silica layer on the zirconia surface to facilitate silane coupling. This includes, but is not limited to, tribochemical silica coating (e.g., CoJet, Rocatec), plasma spraying, glass fusing (sintering of porcelain layers), and internal coating or “Glaze-On” techniques.

### 2.6. Quality Assessment of References

Consistent with the objectives of a scoping review, a formal critical appraisal of individual sources of evidence was not conducted, as the goal was to map the available evidence irrespective of its methodological quality. Nevertheless, an extensive review of the data was conducted to fulfill the study’s purpose.

## 3. Results

### 3.1. Included Studies

**Figure 3:**
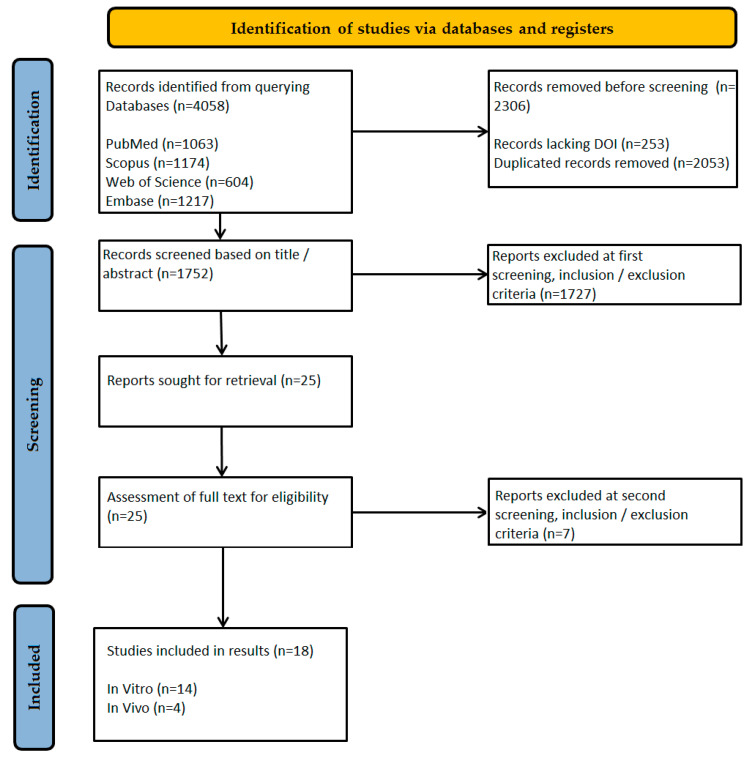
Flow diagram of the study selection process according to PRISMA ScR guidelines.

The initial scoping search identified 4058 records across four databases. After deduplication and filtering, 1752 studies were screened based on their titles and abstracts. In accordance with PRISMA ScR guidelines, 25 full-text articles were evaluated for eligibility, with 18 ultimately meeting the inclusion criteria (see Figure 3).

### 3.2. Outline of Included Studies

Of the 18 studies included, 14 were in vitro investigations validated through laboratory bond strength tests (such as shear or push-out tests), while the remaining 4 were in vivo studies (including 2 prospective cohorts, 1 randomized controlled trial, and 1 retrospective study), featuring diverse experimental designs. Consequently, the final set of studies exhibited significant methodological heterogeneity (Table 2).

Most of the evidence was derived from in vitro studies (*n* = 14), though the inclusion of such diverse experimental approaches provides a realistic view of this study’s goals. As opposed to systematic reviews that aim to address precise research questions, scoping reviews aim to map all available evidence broadly, incorporating various study designs. The key findings from the selected studies are extracted in Table 3.

### 3.3. Overview of Key Findings

The evidence from the included studies was synthesized and is presented herein according to the core objectives of this review: the efficacy of surface treatments, the performance of cementation protocols, and the long-term durability of the resulting bonds. Data on shear bond strength (SBS), failure modes, and clinical outcomes are integrated into this narrative.

It is important to note that the key evidence is based predominantly on in vitro findings, which represents a significant limitation as such studies cannot fully replicate the complex biochemical and biomechanical conditions of the oral environment. Furthermore, the widespread use of shear bond strength tests, while common, is criticized for generating uneven stress distribution at the bonding interface, which may limit the ability to evaluate the true adhesive performance and predict clinical behavior [75]. This issue is further addressed in the discussion section.

#### 3.3.1. Surface Treatments

Surface pretreatment is a fundamental prerequisite for achieving reliable adhesion to zirconia. The evidence consistently demonstrates that micromechanical and chemomechanical interventions significantly outperform untreated surfaces. Air abrasion, particularly with silica-coated alumina particles (tribochemical silicatization) as exemplified by the Rocatec™ system, was found in some studies to increase SBS compared to non-abraded controls [59]. This process is intended to enhance surface roughness and energy, facilitating micromechanical interlocking and providing a silica-rich surface for chemical coupling.

The efficacy of air abrasion was further supported by clinical outcomes. Malgaj et al. reported that nanostructured alumina coating, an advanced air abrasion technique, achieved a clinical survival rate of 93.8% for resin-bonded fixed dental prostheses (RBFDPs), which was comparable to the 86.7% survival rate of conventional airborne-particle abrasion with 50-μm alumina, with no statistically significant difference between the two methods [61]. Beyond mechanical treatments, the critical issue of salivary contamination during clinical try-in procedures was effectively addressed by Kim et al., who demonstrated that a protective ethyl cellulose coating successfully prevented contamination and preserved bond strength, outperforming methods that involved cleaning after contamination had occurred [62].

#### 3.3.2. Cementation Protocols

The choice of cementation protocol, specifically the use of chemical primers, is paramount to achieving high bond strength. Studies uniformly reported that unprimed zirconia yields low and highly variable bond strengths, with SBS values ranging from as low as 2.52 MPa to 33.15 MPa. The application of universal primers containing functional monomers dramatically and significantly improved these outcomes, elevating SBS values to a range of 21.80 to 57.20 MPa [67].

The most effective chemical agent identified across multiple studies was the monomer 10-methacryloyloxydecyl dihydrogen phosphate (MDP). Primers with MDP, such as Clearfil Ceramic Primer Plus, consistently yielded the highest and most durable bond strengths, maintaining efficacy after artificial aging through thermocycling, with reported SBS values exceeding 40 MPa [63]. The superiority of MDP-based primers was also confirmed in the context of endodontic posts, where Z-Prime Plus significantly increased the push-out bond strength of zirconia posts to root dentin [74]. Furthermore, the choice of resin cement itself influences outcomes; Clearfil SA Luting Cement outperformed Panavia F in one study [74], and RelyX Unicem 2 demonstrated significantly higher SBS than MaxCem Elite in another [59], highlighting that the primer–cement combination is a critical factor.

#### 3.3.3. Long-Term Durability

The long-term performance of zirconia bonds is influenced by material composition, manufacturing techniques, and the ability to withstand aging. A key finding is that zirconia’s yttria content directly impacts resin bond stability. Hansson et al. found that 3Y-TZP exhibited the highest SBS after 6 months of water storage (28.98 MPa), which was significantly greater than the bond strengths observed for 4Y-PSZ and 5Y-PSZ (14.35 MPa and 16.05 MPa, respectively) [66]. This trend was corroborated by Suliman et al., who reported that higher yttria content facilitated easier debonding, with Er:YAG laser removal time for 5 mol% Y-TZP (4.03 min) being significantly shorter than for 3 mol% Y-TZP (12.46 min) [69].

The durability of the zirconia–veneer ceramic bond is also affected by manufacturing techniques. Zandinejad et al. determined that milled zirconia substrates provided a superior bond to porcelain (SBS 1.38 MPa) compared to additively manufactured zirconia (SBS 0.68 MPa), a difference that was further exacerbated by thermocycling [64]. While veneering techniques (heat-pressing vs. hand-layering) showed comparable bond strengths [65], the coloring process itself can be detrimental; Celik et al. reported that immersing zirconia in coloring liquid for extended durations (120 s) significantly reduced the SBS of veneering ceramic compared to precolored zirconia [58].

Clinically, the long-term durability of properly bonded zirconia prostheses is excellent. Kern et al. reported a 15-year survival rate of 97.3% for cantilever zirconia RBFDPs, with a retention rate of 82.3% despite some debonding events, the majority of which were successfully rebonded [71]. This outstanding performance is supported by shorter-term studies, such as Yazigi & Kern, who observed a 100% survival rate over a mean period of 53 months [60]. The clinical performance of zirconia RBFDPs was also found to be statistically equivalent to direct composite alternatives over a 3-year period [70].

### 3.4. Summary of Key Findings About Zirconia-Based FDP Restorations: Parameters and Performance

Table 4 summarizes key findings across major parameters, including primers, surface treatments, veneering techniques, porcelain-zirconia bonding, coloring zirconia, thermal cycling and firings, bioceramic cements, fatigue resistance, clinical RBFDP survival rates, and aging effects related to yttria content.

## 4. Discussion

**Figure 4:**
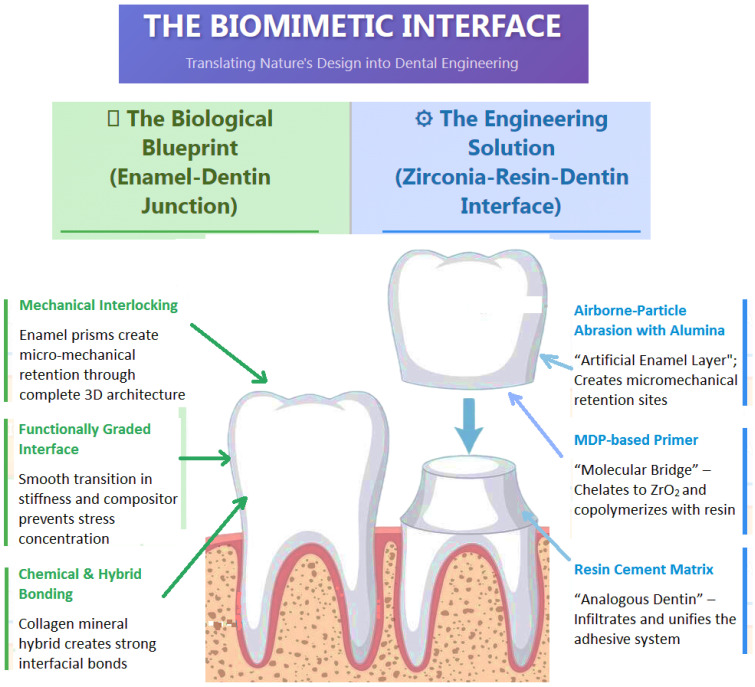
Depiction of a Biomimetic Hybrid Interface.

This scoping review synthesizes evidence on zirconia bonding protocols, cementation strategies, manufacturing techniques, and clinical performance. The following discussion contextualizes key findings, addresses prevailing controversies, and highlights translational implications through a biomimetic lens. The figure below represents a conceptual diagram of analogous biomimetic bonding interfaces to visually articulate the conceptual framework of a “biomimetic lens” (Figure 4).

### 4.1. Surface Pretreatments: Efficacy and Trade-Offs

*Tribochemical Silica Coating (Rocatec™/CoJet)*

Franz et al. (2021) demonstrated superior bond durability with tribochemical silica coating compared to alumina abrasion in their in vitro study, attributed to micromechanical retention and chemical bonding via silica deposition [59]. This process aims to create a biomimetic, silica-rich “artificial enamel” layer on the zirconia surface, making it amenable to conventional silane coupling, a strategy that successfully mimics the bonding protocol for natural silica-based ceramics. This aligns with Prochnow et al. (2025), who reported higher bond strength for 3Y-TZP after CoJet treatment, though translucent zirconias (4Y/5Y-PSZ) showed reduced performance post-aging [76]. Nevertheless, it is paramount to temper the interpretation of these findings. The claim of silica coating superiority often relies on in vitro evidence, and the clinical relevance of silica coating remains debated. Some authors argue that silica particles may not remain firmly embedded in the zirconia surface over the long term, questioning the method’s durability [77]. Furthermore, high clinical failure rates have been reported for resin-bonded FDPs using this method in some studies [78]. Unlike airborne-particle abrasion with alumina, which has been extensively evaluated in long-term clinical trials, robust clinical data for tribochemical silica coating are still lacking. Therefore, it should not be unequivocally stated as a superior method for everyday clinical practice. Quigley et al. (2021) caution that clinical data supporting tribochemical coatings over conventional abrasion remain limited, particularly for monolithic restorations [79].

*Nanostructured Alumina Coating (NAC)*

Malgaj et al. (2023) found comparable clinical retention between nanostructured alumina and airborne-particle abrasion for high-translucency zirconia (5Y-PSZ), mitigating strength degradation risks [61]. This is critical given Wang et al. (2024)’s emphasis on yttria content’s impact on zirconia’s mechanical properties [80]. However, McLaren et al. (2023) noted that nanostructured coatings may not universally enhance bond strength, as substrate composition (e.g., yttria-stabilized vs. cubic-phase zirconia) influences outcomes [81]. In contrast, Malgaj et al. (2021) reported statistically comparable bonding efficacy across 3Y, 4Y, and 5Y zirconia, helping to balance the perspective on the efficiency of NAC [82].

*Ethyl Cellulose Coating*

Kim et al. (2022) showed ethyl cellulose coatings effectively prevent salivary contamination, restoring bond strength to the baseline [62]. While promising for trial placements, Blatz et al. (2018) and Han et al. (2022) highlight that such coatings may complicate definitive cementation if not thoroughly removed, risking interfacial voids [83,84].

### 4.2. Cementation Chemistry: MDP’s Dominance and Alternatives

*MDP-Based Primers/Resins*

Yagawa et al. (2018) and Steiner et al. (2020) reported hydrolytic stability (>40 MPa) with MDP-containing systems (e.g., Panavia V5) [57,63]. The phosphate group in MDP chelates to zirconia’s surface oxides, while the methacrylate group co-polymerizes with the resin cement. This creates a “molecular bridge” or a synthetic protein-like tether, analogous to how collagen fibers in dentin form a hybrid layer with resin monomers, representing a form of molecular biomimicry [85,86,87]. Shokry et al. (2022) corroborate this, showing Panavia V5 outperformed Duo-Link Universal [88]. However, Guilardi et al. (2022) found that MDP’s efficacy depends on aging conditions, and thermocycling reduced bond strengths by 15–20%, suggesting long-term durability requires combined mechanical and chemical retention [89].

*Bioceramic Cements*

Dandoulaki et al. (2019) noted bioceramic cements underperformed versus glass ionomers (2.52–5.23 MPa vs. 4.20–6.61 MPa) [73]. Conversely, Aziz & El-Mowafy (2023) reported 100% 5-year survival with self-adhesive resins (RelyX Unicem 2, Panavia SA), questioning the need for bioactive cements in non-carious substrates [90].

### 4.3. Manufacturing and Veneering: Digital vs. Traditional

*Milled vs. Additive-Manufactured (AM) Zirconia*

Zandinejad et al. (2025) observed 2x higher porcelain bond strength in milled vs. AM zirconia, attributed to density variations in AM parts [64]. Huang et al. (2024) suggest AM process optimization (e.g., laser parameters) could bridge this gap, but current clinical data are lacking [3].

*Digital Veneering*

Sim et al. (2016) reported higher bond strength with digital veneering (28.29 MPa vs. 17.21 MPa), likely due to standardized ceramic application and reduced voids [72]. However, Utar et al. (2023) found conventional layering (e.g., IPS e.max Ceram) yielded superior flexural strength versus digitally veneered zirconia, highlighting a trade-off between precision and mechanical resilience [91].

### 4.4. Aging and Clinical Translation: Bench-to-Bedside Gaps

Yazigi & Kern (2022) correlated in vitro bond strengths >30 MPa with 5-year success rates >95% [60]. However, Elshiyab et al. (2017) noted that aqueous aging protocols often fail to replicate oral conditions (e.g., dynamic loading, pH cycling), potentially overestimating clinical performance [92].

### 4.5. Limitations of This Scoping Review

While this review maps the current evidence on bonding performance of zirconia-based FDPs restorations, its limitations must be acknowledged. The scope was restricted to English-language literature from the past decade, and the evidence base is predominantly derived from in vitro studies (14 of the 18 included studies), which inherently limits the direct extrapolation of findings to clinical outcomes due to the simulation rather than replication of aging, occlusal forces, and the intraoral environment. A significant constraint is the profound heterogeneity in testing protocols, where discrepancies between shear bond strength and microtensile tests complicate direct comparisons (e.g., Zandinejad et al. vs. Steiner et al.), underscoring an urgent need for standardized methodologies such as those outlined in ISO 29022 [57,64,93]. Furthermore, existing in vitro aging models are often incomplete; for instance, the work of Guilardi et al. (2022) demonstrates that fatigue cycling is a critical factor in resin-zirconia interface failure, a variable not consistently incorporated [89]. Most notably, a substantial gap exists in long-term clinical data, as evidenced by sparse evidence for monolithic zirconia crown survival beyond 10 years (Quigley et al., 2021), despite more robust 15-year data available for resin-bonded fixed dental prostheses (Kern et al., 2025) [71,79] These limitations primarily reflect gaps in the current primary literature rather than methodological flaws in the review itself, highlighting specific areas where future research must focus to strengthen clinical translation.

### 4.6. Future Directions

Research Priorities:

- Standardized aging protocols: Incorporate more rigorous aging protocols to better simulate long-term oral conditions, such as 37,500 thermocycles to simulate thermal stress, 150 days of water storage for hydrolysis, and 1.2 million load cycles to approximate five years of clinical service [94,95].
- AM zirconia optimization: Improve interfacial bonding through surface functionalization (e.g., laser patterning) [96,97,98].
- RCTs on novel primers/lasers: Compare zirconia primers (e.g., Z-Prime Plus) and Er:YAG debonding efficacy in clinical settings [99].
- Bio-inspired Interfacial Design: Future research should prioritize the development of truly biomimetic interfaces. This includes investigating functionally graded primers that create a stiffness gradient between zirconia and dentin, mimicking the EDJ. Furthermore, exploring bio-adhesive concepts, such as synthetic polymers inspired by mussel adhesive proteins (e.g., catechol-containing polymers), could lead to novel primers with superior binding to zirconia in the wet oral environment [100,101].

### 4.7. Clinical Recommendations

1. For High-Strength Zirconia (3Y-TZP): According to large clinical studies, airborne-particle abrasion (APA) with alumina (e.g., 50-μm, at low pressure) combined with an MDP-based monomer (e.g., Panavia V5) remains the clinically validated gold standard due to its extensive long-term success data [77]. Tribochemical silica coating + MDP cement shows high bond strengths in vitro and can be considered a promising alternative, but its clinical validation is less robust, and it should not yet be viewed as a superior replacement for APA.
2. For Translucent Zirconia (5Y-PSZ): Nanostructured alumina or low-pressure airborne abrasion to minimize strength loss [61,69] (Malgaj et al., 2023; Sulaiman et al., 2024).
3. For AM Zirconia: Prioritize milled designs until AM process optimization is validated [3] (Huang et al., 2024).

## 5. Conclusions

While advancements in zirconia bonding are significant, translational challenges persist. Future research must standardize testing, incorporate multifactorial aging, and prioritize long-term clinical trials, particularly for monolithic and AM zirconia. This combined approach achieves the hybrid mechanical–chemical adhesion found in natural biological interfaces best.

- Clinical Recommendations: Airborne-particle abrasion with alumina + MDP-based resin cements remains the gold standard with the strongest clinical evidence. A tribochemical silica coating combined with MDP is a promising laboratory strategy but requires more robust long-term clinical validation before it can be universally recommended
- Research Gaps: Standardized aging protocols, bio-inspired interfacial designs, and RCTs comparing novel lasers/primers are needed.

Combined mechanical–chemical approaches yield the most durable bonds for zirconia restorations. Future studies should prioritize clinical validation, standardized testing, and learning from biological blueprints to create the next generation of adhesive interfaces.

## Figures and Tables

**Table 1:** Eligibility Criteria with Rationale.

Category	Inclusion Criteria	Exclusion Criteria	Rationale
Study Design	Comparative studies (in vitro with aging protocols, prospective/retrospective clinical studies, RCTs). Systematic reviews (for background only).	Non-comparative studies; opinion papers; editorials; conference abstracts without full data.	Ensures direct comparison of bonding strategies; excludes low-evidence sources.
Interventions	Clinically applicable and cost-effective surface treatments (e.g., air abrasion with Al_2_O_3_, tribochemical silica coating, MDP-containing primers) and resin cements (e.g., MDP-based, self-adhesive). Standard aging protocols (thermocycling, water storage).	Studies without explicit bonding protocols or controls (e.g., no primer/cement details).	Focuses on pre-treatment methods and materials that are widely available and practical for clinical use and represent the current standard of care or near-future options for general dentistry.
Outcomes	Quantitative bond strength (shear/microtensile, MPa); clinical survival/debonding rates; failure mode analysis.	Qualitative outcomes (e.g., subjective assessments only); studies lacking bond strength/aging data.	Focuses on measurable, clinically relevant adhesive performance.
Applications	Zirconia crowns, fixed dental prostheses (FDPs), resin-bonded FDPs (RBFDPs).	Non-zirconia materials (e.g., lithium disilicate); temporary/provisional prostheses.	Maintains focus on permanent zirconia restorations with adhesive challenges.
Publication	Peer-reviewed articles (2015–June 2025); English language.	Non-peer-reviewed (theses, patents); non-English (without translation); pre-2015.	Ensures methodological rigor and relevance to current bonding technologies.

**Table 2:** Incorporation of Different Research Designs.

Author (Year)	Study Type	Study Design	Sample Size (*n*)	Variables Tested
Steiner et al. (2020) [57]	In vitro	Controlled experiment	170 specimens	Resin cements (7 types), priming protocols (universal/system-specific)
Celik et al. (2020) [58]	In vitro	Comparative	64 specimens	Coloring duration (30s/60s/120s), precolored vs. uncolored zirconia
Franz et al. (2021) [59]	In vitro	Two-part experiment	Not specified	Air abrasion (Rocatec™), glaze-on, primers (Clearfil, Monobond)
Yazigi and Kern (2022) [60]	In vivo (prospective cohort)	Clinical follow-up	27 restorations (21 patients)	Survival rate, debonding events (mean 53 months)
Malgaj et al. (2023) [61]	In vivo (RCT)	Randomized controlled trial	31 restorations (27 patients)	Nanostructured alumina vs. airborne abrasion (double-blind)
Kim et al. (2022) [62]	In vitro	Controlled experiment	72 specimens	Cleaning methods (ultrasonic, zirconia cleaner, ethyl cellulose)
Yagawa et al. (2018) [63]	In vitro	Comparative	308 specimen pairs	Primers (7 types), luting agents (Panavia V5, Opaque)
Zandinejad et al. (2025) [64]	In vitro	Comparative	Not specified (*n* = 10/group)	Milled vs. AM zirconia, surface treatments
Teng et al. (2024) [65]	In vitro	Comparative	42 specimens	Hand-layering vs. heat-pressing, residual stress
Hansson et al. (2024) [66]	In vitro	Comparative	131 specimens	3Y-PSZ vs. 4Y-PSZ vs. 5Y-PSZ, aging (6 months)
Cadore-Rodrigues et al. (2021) [67]	In vitro	Controlled experiment	Not specified	Grinding protocols (fine, extra fine), polishing
Hensel et al. (2022) [68]	In vitro	Controlled experiment	600 specimens	Firing cycles (2–10), thermocycling
Suliman et al. (2024) [69]	In vitro	Comparative	40 crowns	3Y/4Y/5Y zirconia vs. lithium disilicate, debonding time
Sato et al. (2024) [70]	In vivo (retrospective)	Multicenter retrospective	45 patients (17 CR-RBFDP, 28 Zr-RBFDP)	Survival rate (3-year follow-up)
Kern et al. (2025) [71]	In vivo (prospective cohort)	Clinical follow-up	328 restorations (258 patients)	15-year survival rate, pontic location effect
Sim et al. (2016) [72]	In vitro	Comparative	50 specimens	Digital veneering vs. conventional methods
Dandoulaki et al. (2019) [73]	In vitro	Comparative	240 specimens (120 dentin, 120 zirconia)	Bioceramic vs. glass ionomer cement
Torabi Ardakani et al. (2015) [74]	In vitro	Comparative	40 teeth (120 segments)	MDP primer (Z-Prime Plus) vs. no primer

**Table 3:** Selected Literature with Key Findings.

Author (Year)	Aging Protocol?	MDP/Silica Comparison?	Key Findings
Steiner et al. (2020) [57]	Yes (water storage + thermocycling)	Yes (MDP vs. non-MDP primers)	Universal and system-specific MDP-based primers significantly improved bond strength (21.8–57.2 MPa) vs. unprimed (2.5–33.2 MPa). Cohesive failures correlated with higher bond strength.
Celik et al. (2020) [58]	No thermocycling reported	No MDP comparison	Precolored zirconia (PCZ) had higher bond strength (31.5 MPa) vs. colored groups (16.6–31.5 MPa). Coloring liquids reduced bond strength.
Franz et al. (2021) [59]	Yes (thermocycling)	Yes (MDP vs. no primer)	Air abrasion (Rocatec™ Soft) + MDP primers (Clearfil Ceramic Primer/Monobond S) yielded highest bond strength. Glaze-on techniques were ineffective.
Yazigi and Kern (2022) [60]	Yes (13–151 months clinical follow-up)	No silica comparison	100% survival rate (mean 53 months) with MDP-based cementation (Panavia V5). Minimal tooth preparation.
Malgaj et al. (2023) [61]	Yes (8.3–37.9 months clinical follow-up)	No MDP comparison	Nanostructured alumina (93.8% survival) performed similarly to airborne abrasion (86.7%).
Kim et al. (2022) [62]	Yes (immediate/short/ long-term aging)	No MDP comparison	Ethyl cellulose coating restored bond strength after contamination (higher than ultrasonic cleaning).
Yagawa et al. (2018) [63]	Yes (5000 thermocycles)	Yes (MDP vs. non-MDP primers)	MDP-containing primers (Alloy Primer, Clearfil Ceramic Primer) showed highest bond strength (32.4–47.8 MPa) post-aging.
Zandinejad et al. (2025) [64]	Yes (thermocycling)	No silica/MDP comparison	Milled zirconia had higher bond strength (1.38 MPa) vs. AM zirconia (0.68 MPa). Surface treatments had no significant effect.
Teng et al. (2024) [65]	No thermocycling	No MDP comparison	Heat-pressed veneering showed comparable bond strength to hand-layering.
Hansson et al. (2024) [66]	Yes (6-month water storage)	Yes (MDP primer used)	3Y-PSZ with MDP primer had highest bond strength (31.8 MPa) vs. 4Y/5Y-PSZ.
Cadore-Rodrigues et al. (2021) [67]	Yes (step-stress fatigue)	No MDP comparison	Grinding reduced fatigue resistance. Polishing had no effect.
Hensel et al. (2022) [68]	Yes (thermocycling)	No MDP comparison	Leucite-free veneering ceramic (ZRT) had highest bond strength.
Suliman et al. (2024) [69]	No thermocycling	No MDP comparison	Debonding time decreased with higher yttria content (3Y: 12.46 min; 5Y: 4.03 min).
Sato et al. (2024) [70]	Yes (3-year follow-up)	No MDP comparison	Similar survival rates for Zr-RBFDPs (91.7%) and CR-RBFDPs (92.3%).
Kern et al. (2025) [71]	Yes (85-month mean follow-up)	No silica comparison	97.3% 15-year survival rate with MDP-based cementation (Panavia).
Sim et al. (2016) [72]	No thermocycling	No MDP comparison	Digital veneering method (28.3 MPa) outperformed conventional methods (17.2–18.9 MPa).
Dandoulaki et al. (2019) [73]	Yes (30-day water storage)	No MDP comparison	Bioceramic cement had lower bond strength (2.5–5.2 MPa) vs. glass ionomer (4.2–6.6 MPa).
Torabi Ardakani et al. (2015) [74]	No thermocycling	Yes (MDP vs. no primer)	MDP-based primer (Z-Prime Plus) improved bond strength (Clearfil SA > Panavia F). Coronal > apical bond strength.

**Table 4:** Summary of Key Findings about Zirconia-Based FDPs Restorations: Parameters and Performance.

Parameter	Key Result	Reference
Primers	Universal primers significantly improve SBS (*p* < 0.05). MDP-based primers most effective.	[57,63,74]
Surface Treatments	Air abrasion > glaze-on. Nanostructured alumina comparable to Al_2_O_3_ abrasion. Ethyl cellulose prevents contamination.	[59,61,62]
Veneering Techniques	Digital veneering > heat-pressed > hand-layered. Heat-press comparable to hand-layering.	[65,72]
Porcelain–Zirconia Bonding	Milled zirconia (1.38 MPa) > AM zirconia (0.68 MPa). Surface treatment (abrasion/liner) had no effect.	[64]
Coloring Zirconia	Precolored zirconia (PCZ) had higher SBS (31.5 MPa) vs. colored (16.6 MPa).	[58]
Thermal Cycling and Firings	Multiple firings reduced bond strength. Leucite-free ceramics showed best thermal stability.	[68]
Bioceramic Cements	Glass ionomer cement (4.20–6.61 MPa) > bioceramic cement (2.52–5.23 MPa). No apatite formation.	[73]
Fatigue Resistance	Grinding inner surfaces reduced fatigue strength (ZR: 733–880 N vs. control 973 N). Polishing had no effect.	[67]
Clinical RBFDP Survival	97.3% at 15 years. No difference between composite and zirconia RBFDPs.	[60,70,71]
Aging and Yttria Content	3Y-PSZ > 5Y-PSZ in bond strength. Higher yttria reduces laser debonding time.	[66,69]

## Data Availability

No new data were created.

## Supplementary Materials

The following supporting information can be downloaded at https://www.mdpi.com/article/10.3390/biomimetics10090632/s1: Supplementary File S1: Prisma ScR Checklist. Supplementary File S2: Search strategy.

## Abbreviations

The following abbreviations are used in this manuscript:

MDP	10-methacryloyloxydecyl dihydrogen phosphate
ZrO_2_	zirconia
MPa	megapascal
FDPs	fixed dental prostheses
EDJ	enamel–dentin junction
3D	three-dimensional
FRC	fiber-reinforced composite
RBFDP	resin-Bonded Fixed Dental Prosthesis
AM	additively manufactured
3Y-TZP	3 mol% yttria-stabilized tetragonal zirconia polycrystal
4Y-PSZ	4 mol% Yttria-stabilized Partially Stabilized Zirconia
5Y-PSZ	5 mol% yttria-partially stabilized zirconia
NAC	nanostructured Alumina Coating
