# Supplementary material for: Bonding Strategies for Zirconia Fixed Restorations: A Scoping Review of Surface Treatments, Cementation Protocols, and Long-Term Durability
Source: Biomimetics (Basel). 2025 Sep 19;10(9):632. doi: 10.3390/biomimetics10090632 (PMC12468000; doi:10.3390/biomimetics10090632)
Supplement: Supplementary file 1 [file biomimetics-10-00632-s001.zip › biomimetics-3862656-Supplementary File S2 - Search.pdf]

Supplementary File S2

Keywords and Subject Headings Used During the Search

**Database**	**Search strategy**
PubMed accessed on July 1, 2025	(<br>("Zirconium"[Mesh] OR "zirconia"[tiab] OR "zirconium oxide"[tiab] OR "zirconium dioxide"[tiab] OR "ZrO2"[tiab] OR "Y-TZP"[tiab] OR "yttria-stabilized zirconia"[tiab] OR "zirconia ceramic"[tiab] OR "zirconia-based"[tiab])<br>AND<br>("Dental Crowns"[Mesh] OR "Crowns"[Mesh] OR "Dental Prosthesis"[Mesh] OR "Dental Restoration, Permanent"[Mesh] OR "dental crown*"[tiab] OR "dental bridge*"[tiab] OR "fixed dental prosthesis"[tiab] OR "FDP"[tiab] OR "zirconia restoration*"[tiab] OR "zirconia crown*"[tiab] OR "zirconia bridge*"[tiab])<br>AND<br>("Dental Bonding"[Mesh] OR "Cementation"[Mesh] OR "Resin Cements"[Mesh] OR "dental bonding"[tiab] OR "bonding strategy"[tiab] OR "adhesive cementation"[tiab] OR "resin cement"[tiab] OR "surface treatment"[tiab] OR "sandblasting"[tiab] OR "tribochemical coating"[tiab] OR "silica coating"[tiab] OR "laser treatment"[tiab] OR "MDP"[tiab] OR "10-MDP"[tiab] OR "primers"[tiab] OR "zirconia bonding"[tiab] OR "shear bond strength"[tiab] OR "microtensile bond strength"[tiab] OR "durability"[tiab] OR "aging"[tiab] OR "thermocycling"[tiab])<br>)<br>AND ("2015/01/01"[Date - Publication] : "2025/06/30"[Date - Publication])<br>AND English[Language]
Scopus accessed on June 30, 2025	TITLE-ABS-KEY (<br>(zirconia OR "zirconium oxide" OR "zirconium dioxide" OR ZrO2 OR "Y-TZP" OR "yttria-stabilized zirconia" OR "zirconia ceramic" OR "zirconia-based")<br>AND<br>("dental crown*" OR "dental bridge*" OR "fixed dental prosthesis" OR FDP OR "zirconia restoration*" OR "zirconia crown*" OR "zirconia bridge*" OR "dental prosthesis" OR "dental restoration*")<br>AND<br>("dental bonding" OR "bonding strategy" OR "adhesive cementation" OR "resin cement" OR "surface treatment" OR sandblasting OR "tribochemical coating" OR "silica coating" OR "laser treatment" OR MDP OR "10-MDP" OR primers OR "zirconia bonding" OR "shear bond strength" OR "microtensile bond strength" OR durability OR aging OR thermocycling OR "cement* bond*")<br>)<br>AND PUBYEAR > 2014<br>AND PUBYEAR < 2026

	AND (LIMIT-TO(LANGUAGE, "English"))
Web Of Science accessed on July 8, 2025	TS=(<br>(zirconia OR "zirconium oxide" OR "zirconium dioxide" OR ZrO2 OR "Y-TZP" OR "yttria-stabilized zirconia" OR "zirconia ceramic" OR "zirconia-based")<br>AND<br>("dental crown*" OR "dental bridge*" OR "fixed dental prosthesis" OR FDP OR "zirconia restoration*" OR "zirconia crown*" OR "zirconia bridge*")<br>AND<br>("dental bonding" OR "bonding strategy" OR "adhesive cementation" OR "resin cement" OR "surface treatment" OR sandblasting OR "tribochemical coating" OR "silica coating" OR "laser treatment" OR MDP OR "10-MDP" OR primers OR "zirconia bonding" OR "shear bond strength" OR "microtensile bond strength" OR durability OR aging OR thermocycling)<br>)<br>AND PY=(2015-2025)<br>AND LA=(English)
Embase accessed accessed on July 3, 2025	('zirconium oxide'/exp OR 'zirconia':ti,ab,kw OR 'zirconium dioxide':ti,ab,kw OR 'zro2':ti,ab,kw OR 'y-tzp':ti,ab,kw OR 'yttria-stabilized zirconia':ti,ab,kw OR 'zirconia ceramic':ti,ab,kw OR 'zirconia-based':ti,ab,kw) AND ('dental crown'/exp OR 'dental prosthesis'/exp OR 'fixed dental prosthesis':ti,ab,kw OR 'fdp':ti,ab,kw OR 'dental crown*':ti,ab,kw OR 'dental bridge*':ti,ab,kw OR 'zirconia restoration*':ti,ab,kw OR 'zirconia crown*':ti,ab,kw OR 'zirconia bridge*':ti,ab,kw) AND ('dental bonding'/exp OR 'tooth cement'/exp OR 'resin cement'/exp OR 'dental bonding':ti,ab,kw OR 'bonding strategy':ti,ab,kw OR 'adhesive cementation':ti,ab,kw OR 'resin cement':ti,ab,kw OR 'surface treatment':ti,ab,kw OR 'sandblasting':ti,ab,kw OR 'tribochemical coating':ti,ab,kw OR 'silica coating':ti,ab,kw OR 'laser treatment':ti,ab,kw OR 'mdp':ti,ab,kw OR '10-mdp':ti,ab,kw OR 'primers':ti,ab,kw OR 'zirconia bonding':ti,ab,kw OR 'shear bond strength':ti,ab,kw OR 'microtensile bond strength':ti,ab,kw OR 'durability':ti,ab,kw OR 'aging':ti,ab,kw OR 'thermocycling':ti,ab,kw) AND [2015-2026]/py AND [english]/lim
